# Virtual versus jaw simulation in inlay preparation preclinical teaching: a randomised controlled trial

**DOI:** 10.1186/s12909-022-03930-6

**Published:** 2022-12-06

**Authors:** Jie Sheng, Congdi Zhang, Zhengkun Gao, Yimin Yan, Yucheng Meng, Shiqi Ren, Bin Liu, Baoping Zhang

**Affiliations:** 1 School of Stomatology Lanzhou University, Lanzhou, 730000 China; 2 Hospital of Stomatology, Lanzhou University, Lanzhou, 730000 China; 3 Gansu Province Key Lab of Maxillofacial Reconstruction and Intelligent Manufacturing, Lanzhou, 730000 China; 4 Gansu Province Clinical Research Center for Oral Diseases, Lanzhou, 730000 China

**Keywords:** Dental education, Virtual simulation, Inlays preparation, Prosthodontics

## Abstract

**Background:**

To investigate the effect of virtual simulation systems on the teaching of inlay experiments and to guide the experimental teaching of tooth preparation.

**Methods:**

Participants in their second semester of the junior year were selected to carry out the unified teaching and evaluation of dental preparation theory. The age varied from 18 to 22 years (19.96 ± 0.70) and the participants were randomly divided into four groups (n = 19) with a similar male-to-female ratio following CONSORT guidelines, including a jaw simulation model training group (Group J), a virtual simulation system training group (Group V), a jaw model training first followed by a virtual system training group (Group J-V), and a virtual system followed by a jaw model training group (Group V-J). The inlay tooth preparation assessment was performed on the extracted teeth. The data were analysed according to the assessment scores by a senior clinician. The subjective feelings of the students towards the system were evaluated using questionnaires.

**Results:**

The second theoretical scores of Group V-J (63.5 ± 2.89) and Group J-V (60.5 ± 3.25) were higher than those of Group V (57.5 ± 3.13) and Group J (58.0 ± 3.67). The experimental scores of Groups J-V and V-J (62.79 ± 2.84; 64.00 ± 2.85) were higher than those of Groups V and J (56.05 ± 3.39; 55.74 ± 2.53). The questionnaire survey illustrated that most students preferred the digital virtual simulation system (perfect assessment: 91.3%, accuracy: 82.6%, satisfaction: 52.2%).

**Conclusion:**

Virtual simulation training can facilitate the teaching effect of tooth preparation in inlay experiments, and the teaching mode of Group V-J was the best. Therefore, this teaching mode is to be popularised.

**Supplementary Information:**

The online version contains supplementary material available at 10.1186/s12909-022-03930-6.

## Introduction

Dental education, based on its special practicality and strong operability, requires qualified preclinical training and assessment of practitioners. Preclinical training can provide timely feedback on the learning level of students and improve their clinical skills and practical ability [1, 2]. Before the emergence of a virtual simulation system, a jaw model has been mainly used for medical experimental teaching and preclinical skills training [3]. Using this model, stomatology undergraduates are trained in basic dental operative skills, such as adjusting the patient's chair position, applying basic dental examination instruments, selecting and applying fulcrums, and performing dental preparation and restoration on jaw models in simulation exercises. However, this method, which is more time-consuming and expensive with few models, cannot simulate the common and difficult cases in the clinical practice of real situations [3–6].

The virtual simulation system can remedy the defects of the jaw model and considerably meet the training requirements. Through the interaction between individuals and the scene generated by computers, the operators can effectively interact with the 3D computer database in real-time, stimulate visual, auditory, tactile, and other sensory modes [7, 8], and carry out learning and training. Practice-based on the virtual simulation system can reduce experimental materials, provide more training models to simulate clinical cases [9, 10], offer new ideas for preclinical training, and open up new opportunities for experimental teaching [11, 12]. Currently, the virtual simulation system is widely used in oral preclinical training teaching. It is widely applied in oral histopathology interactive digital slice reading, nerve block anesthesia teaching in oral surgery, the experimental teaching of tooth preparation and restoration, and various cavity preparation and pulpotomy treatment for dental pulp diseases [11, 13]. Further, it has been applied for the digital treatment of periodontal system sequence and the experimental teaching of the dental implants [14]. A representative study has evaluated the effect of using virtual reality (VR) in the endodontic curriculum for teaching root canal anatomy to third-year undergraduate students. It has been observed that VR has considerable advantages over three-dimensional reconstructions and two-dimensional radiographs when teaching root canal anatomy, and students are pleased with the development [15]. Additionally, studies on the contribution of VR to the conventional analogic training environment have revealed the complementarity of conventional techniques and VR in the learning of dental students [16]. Zafar S et al. [17] have found that participants feel more comfortable with Simodont for practical exercises and have suggested that Simodont can be used as an adjunct in training dental students for preclinical paediatric dentistry restorative exercises.

An inlay restoration is an aesthetic restoration embedded in the cavity of the teeth and is tailored for the teeth to restore the morphology and function of the defective teeth. It is particularly used for the tooth with significantly short occlusion–gingiva distance and where a filling is difficult because of the poor retention form and resistance form; thus, inlay restoration has broad application prospects in the clinic [18, 19]. However, owing to the high technical sensitivity of the requirements for tooth preparation in this process, the majority of doctors do not fully understand the technique. Additionally, stomatology students do not fully understand this technique in the experimental teaching of tooth preparation for inlay restoration. Therefore, stomatology students need preclinical inlay preparation training. At present, there is no relevant research report on its application in the experimental teaching of dental preparation for inlay restoration. In this study, we aim to determine if preclinical experimental teaching can be used in a mode that combines virtual simulation with reality and if they will complement each other. Additionally, we investigate if the sequence and combination of the two training methods will be of practical significance in experimental teaching.

Herein, we applied the virtual simulation system in the prosthodontics experimental teaching through randomised controlled trials and evaluated the role of the virtual system in the experimental teaching of dental preparation for inlay restoration. A questionnaire survey was conducted to assess the students' attitude and satisfaction with the virtual system and the effectiveness of the system in inlay-preparation-laboratory teaching, with the aim of guiding the experimental teaching of dental preparation. This will provide new ideas for expanding and reforming traditional oral preclinical experimental teaching and improving its clinical accessibility.

## Methods

This study was approved by the Ethics Committee of the School of Stomatology Lanzhou University (No. LZUKQ-2019-25), and all students voluntarily participated in the study and signed the informed consent form (Registration number: ChiCTR2200057760, date: 16/03/2022). All experimental protocols involving human subjects were conducted following the Declaration of Helsinki (2013) [20].

### Participants

Seventy-six undergraduates of the school of stomatology in their second semester of the junior year were chosen as the study objects to undertake the unified teaching and assessment of dental preparation theory. The age varied from 18 to 22 years, with a roughly 1:1 ratio of male to female (Mean ± SD, 19.96 ± 0.70). And there was no significant difference in age (*P >* 0.05). Eighty-two undergraduates were classified into two groups (male/female) according to gender. Thereafter, 38 students were randomly selected in each of the two packets using a random-number table consisting of 76 participants, which can scientifically draw random sampling. Notably, the students did not learn periodontology and tooth preparation before the experiment to achieve the same starting point.

### Study procedure

#### Theoretical teaching and grouping

**Fig. 1:**
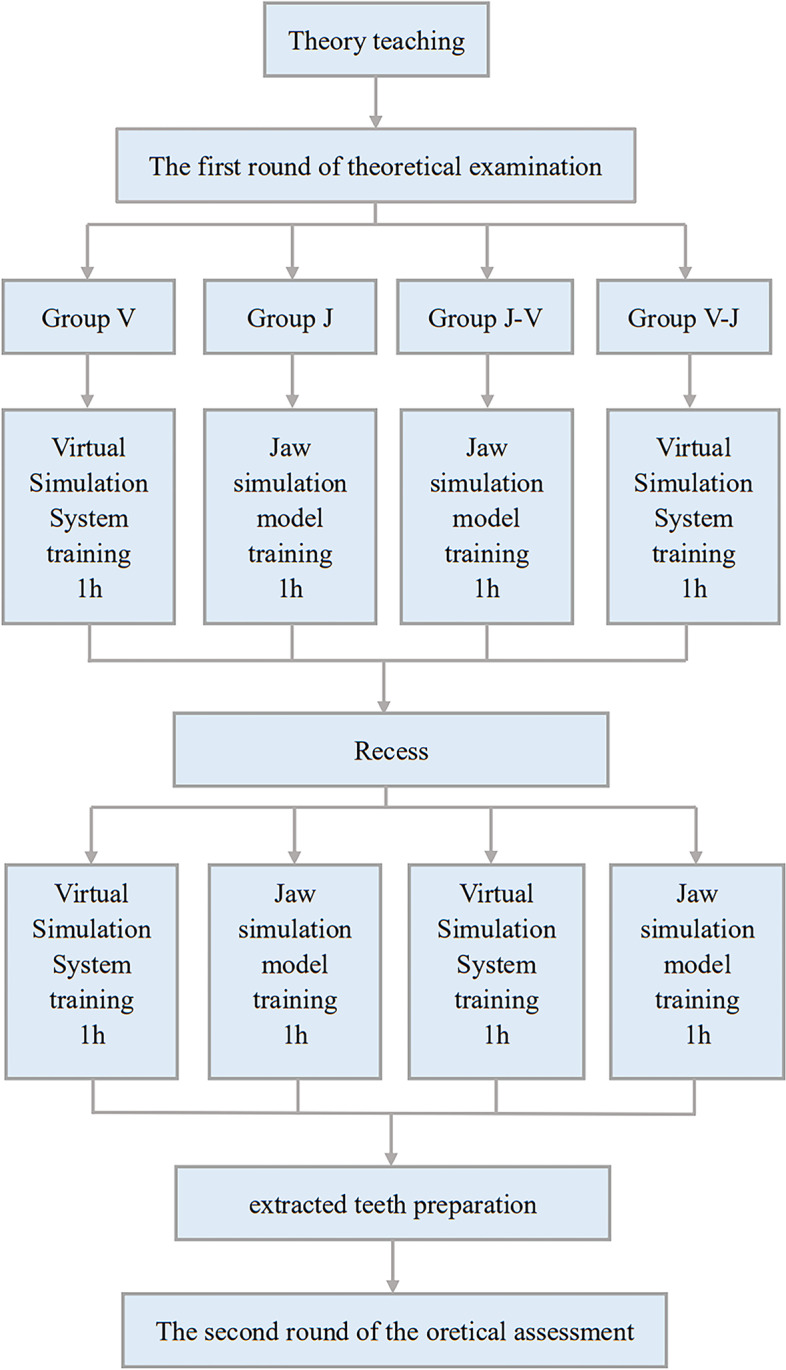
Flow chart

The experimental process is shown in Fig. 1. Participants received a theoretical lecture on tooth preparation for inlay restoration, and afterwards, the first theoretical examination was implemented. The objects were randomly split into 4 groups, each with 19 volunteers, that is, the virtual simulation system training group (Group V), jaw model training group (Group J), jaw model training first followed by virtual simulation system training group (Group J-V), and virtual simulation system training first followed by jaw model training group (Group V-J).

#### Dental preparation training

**Fig. 2:**
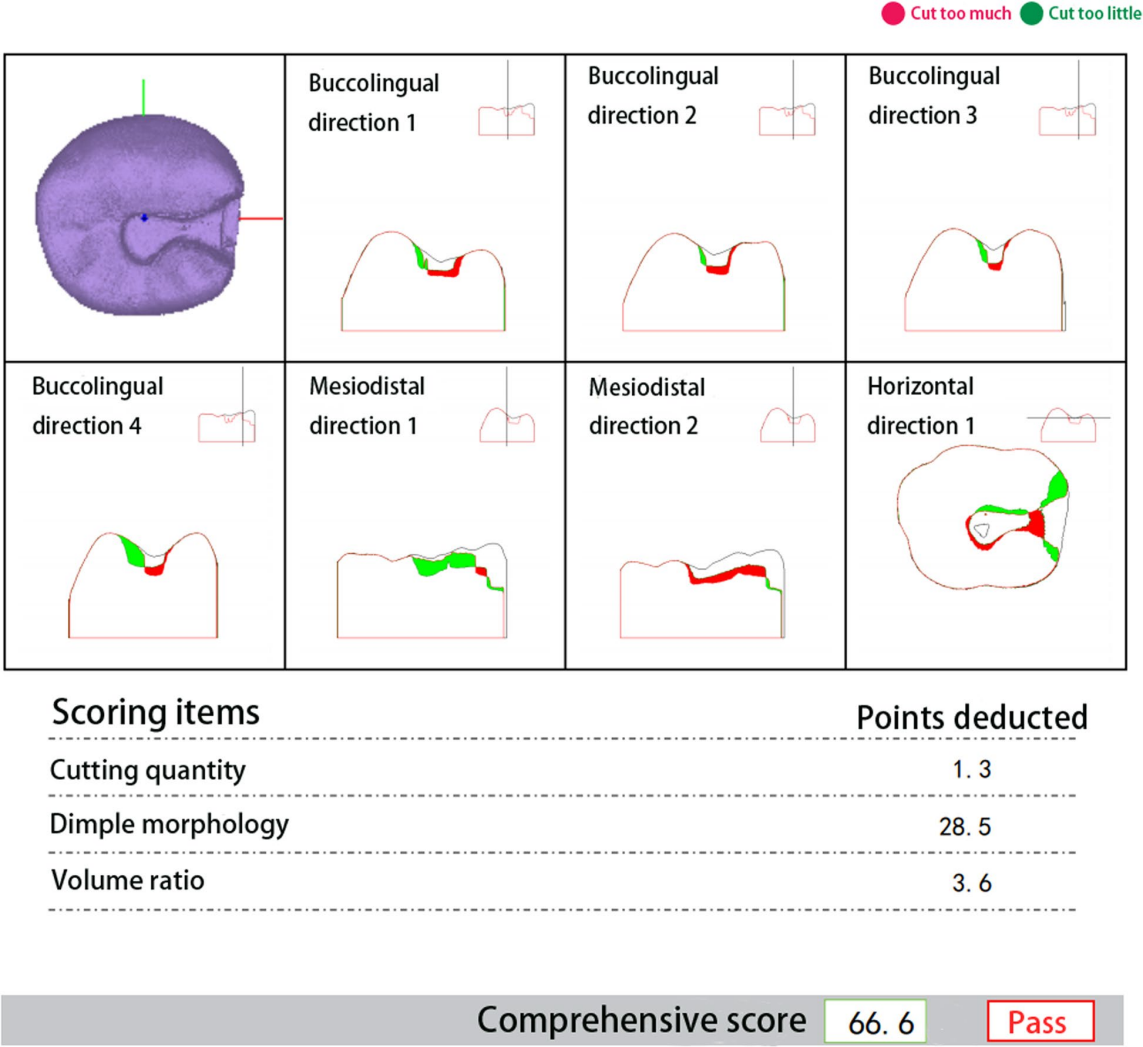
The report of a subject after preparation and scanning

Depending on the grouping, the virtual simulation system (UniDental-MU01, UNIDRAW, China) and the jaw model (Type II, NISSIN, Japan) were employed for training. Each object using the virtual system received the scoring report after completing the preparation for every exercise. An analysis report of one of the subjects in the exercise is shown in Fig. 2. Groups V and J received 2 hours of training, while Groups J-V and V-J received 1 hour of training in a virtual system and jaw model, respectively. The training was divided into two phases.

#### Dental preparation examination

**Fig. 3:**
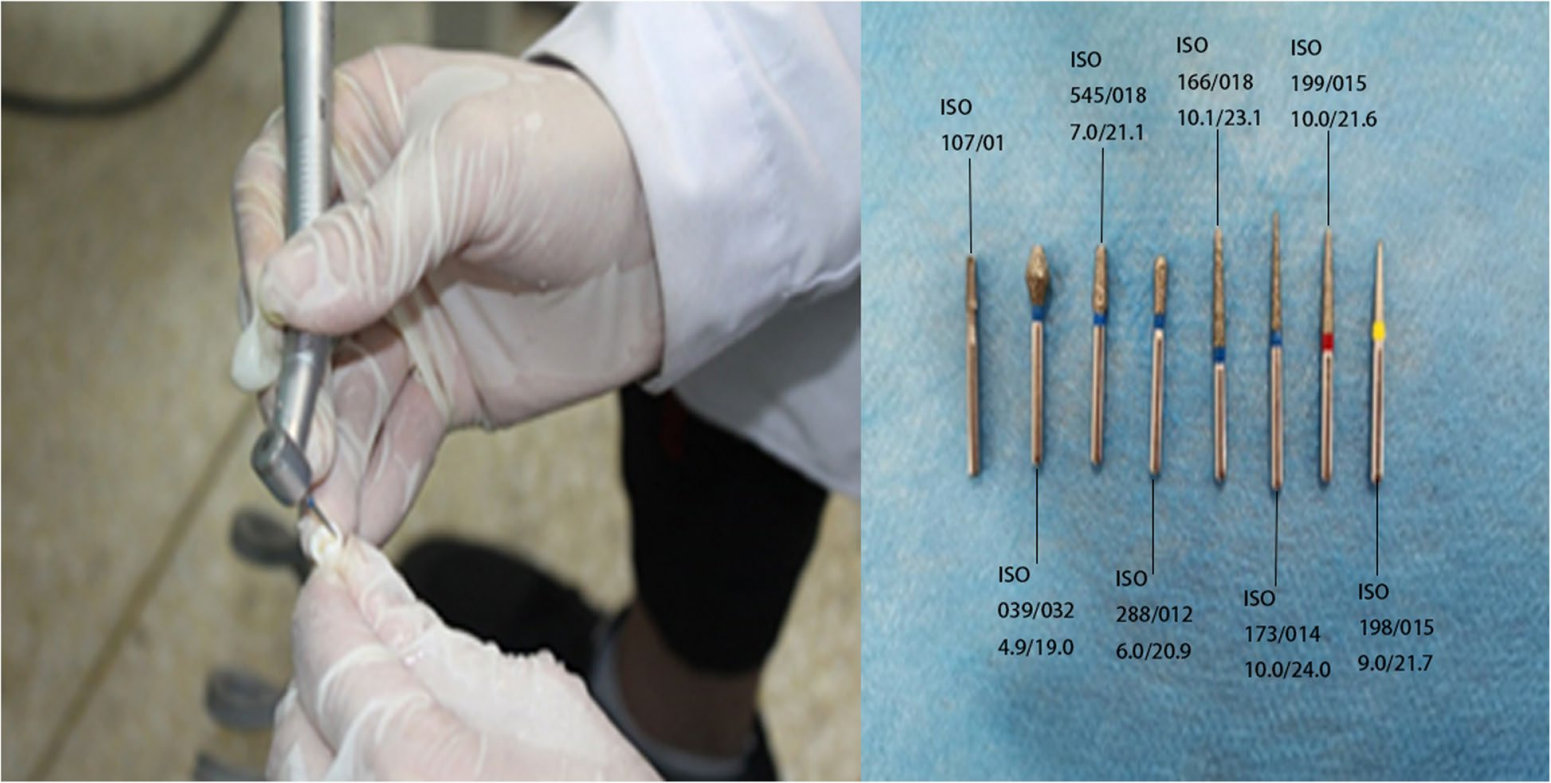
Examination process of extracted teeth and dental bur provided by examination

**Fig. 4:**
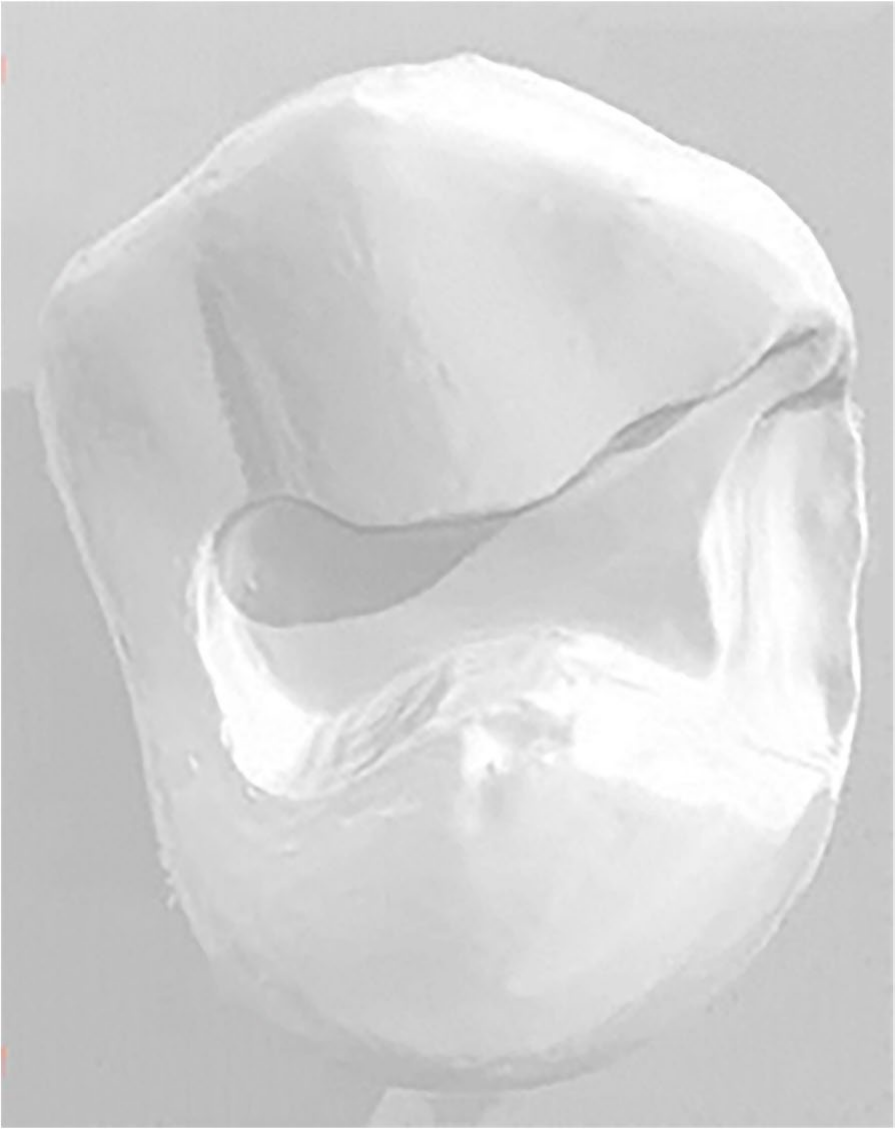
Teeth preparation form obtained by scanning technology

After the first hour of training, participants took a 10-minute break before the next hour of training. Thereafter, the four groups underwent a dental preparation examination of the extracted first premolar (Fig. 3), and the results of the preoperative preparation, operative procedure and dental preparation for the extracted teeth were scored by a senior clinical faculty. A scanning technique was employed for a clear form of dental preparation to observe the effect of dental preparation (Fig. 4); the scoring standard is shown in Table 1. Then the second theoretical test was conducted.

#### Questionnaire survey

A questionnaire was given to all the participants after the examination. The questionnaire surveyed the degree of satisfaction and accuracy of the study. Each item was rated, including ‘Disagree’, ‘Partly agree’, ‘Agree’, and ‘Strongly agree’ (Table 3).

### Statistical analysis

Using IBM SPSS statistics software (version 26), One-way ANOVA was performed on the theoretical scores (twice) and operational assessment scores for each group, and two paired-sample *T*-tests were adopted on the theoretical scores. Afterwards, subjects subjectively evaluated the virtual simulation system using a Likert scale.

## Results

### Results of the first theoretical assessment

**Fig. 5:**
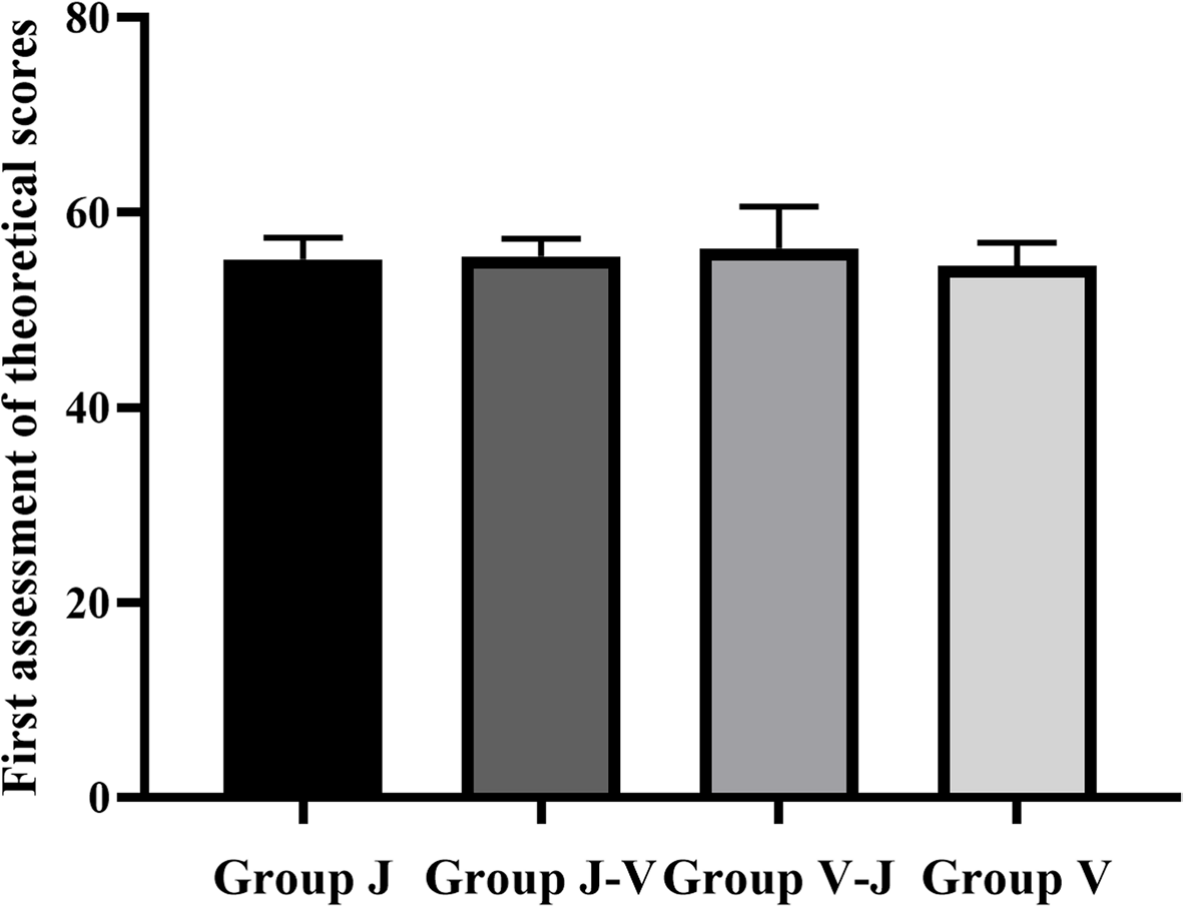
First assessment of theoretical scores

An ANOVA was performed, and it showed no significant difference in the average scores among the V, J, V-J, and J-V groups (54.5 ± 2.35, 55.2 ± 2.21, 56.3 ± 4.27, 55.5 ± 1.77, respectively) (P > 0.05) (Figure 5), indicating the same degree of students’ learning ability after the theoretical teaching.

### Performance of the second theoretical examination

**Fig. 6:**
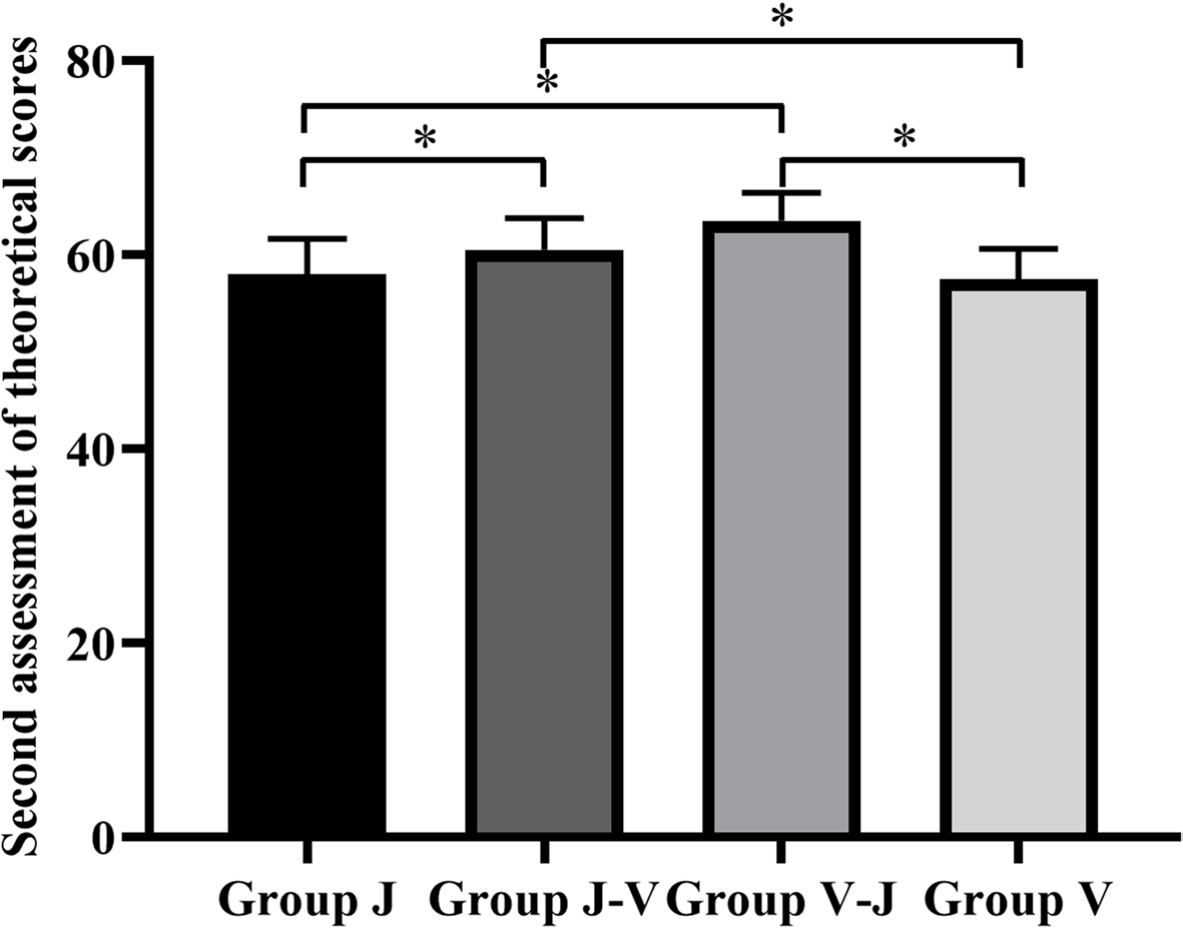
Second assessment of theoretical scores

As shown in Fig. 6, the results of Group V-J (63.5 ± 2.89) and Group J-V (60.5 ± 3.25) were higher than those of Group V (57.5 ± 3.13) and Group J (58.0 ± 3.67), among which Group V-J had the best results (P < 0.05) under the ANOVA method. In addition, there were significant differences among the scores of Groups V, V-J, and J-V (*P* < 0.05). Further, there were significant differences among the scores of Groups J, V-J, and J-V (*P* < 0.05). It is not statistically meaningful among other groups (P > 0.05).

**Fig. 7:**
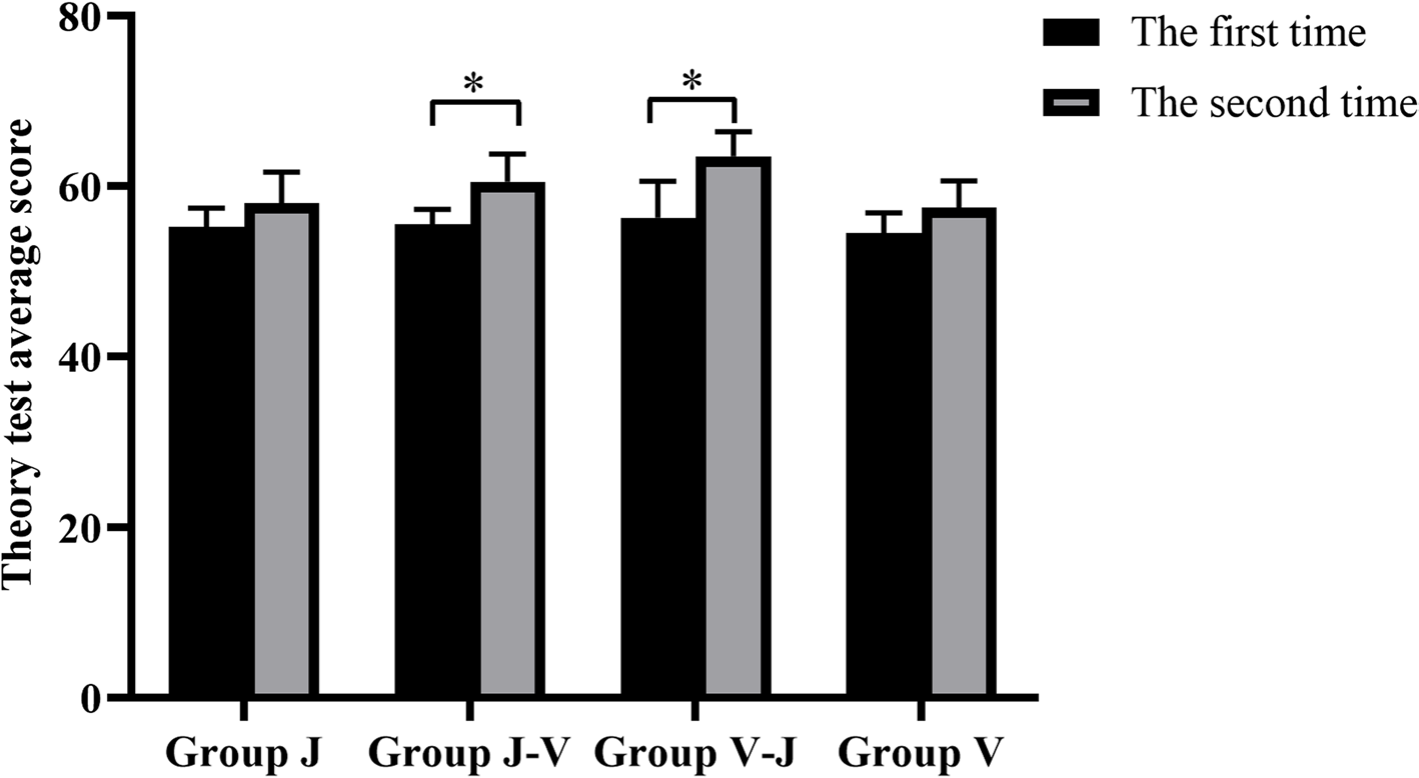
Comparison of two theoretical assessment results

After the operation training, improvement was shown in the scores of the second theoretical examination of each group in comparison with the first (Fig. 7), as determined by a paired-sample *T*-test, and the scores of Groups V-J and J-V were significantly improved (*P* < 0.0001). The operation training significantly promoted the students' mastery of theoretical knowledge, and the teaching effect of the combination of the virtual simulation system and traditional simulation head model was better in the same period.

### Examination results of extracted teeth preparation

**Fig. 8:**
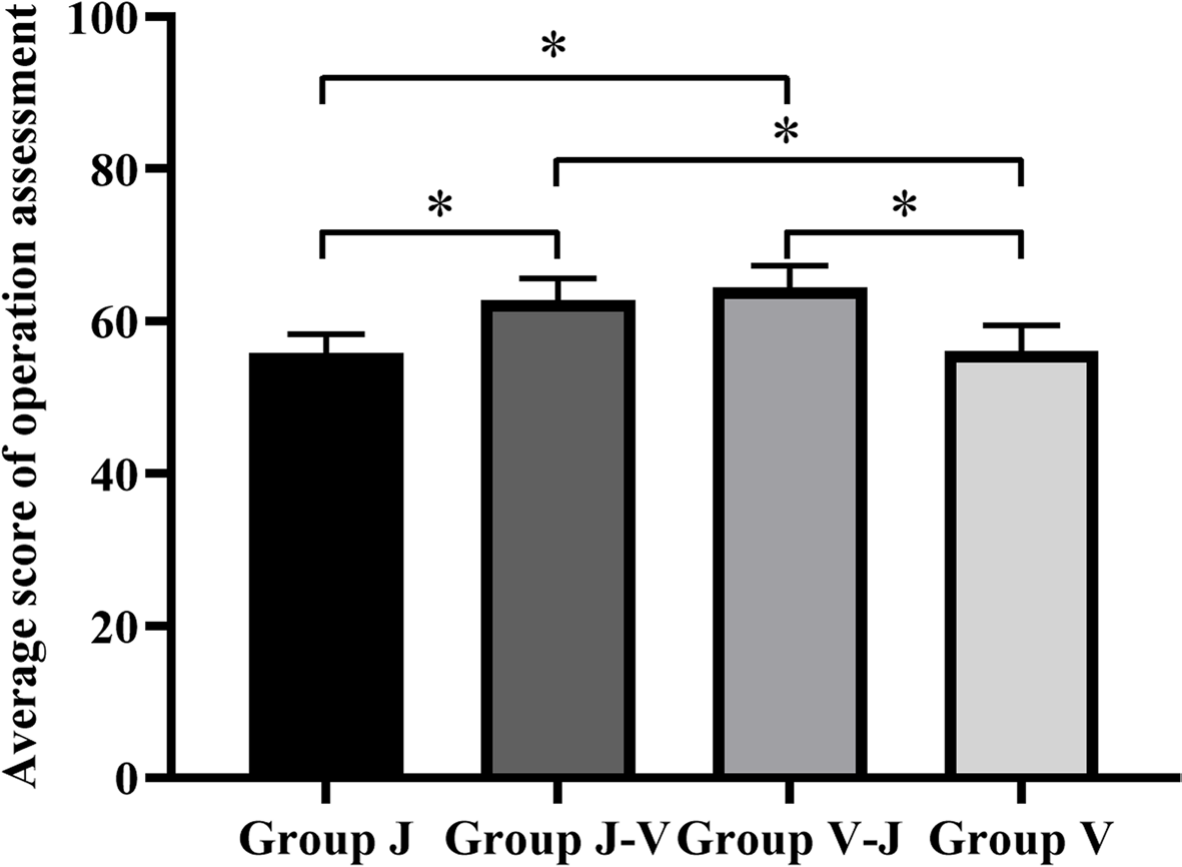
Average score of operation assessment of each group

The operational scores are shown in Table 2. As shown in Fig. 8, the ANOVA among the groups showed that the training effect of Groups J-V (62.79 ± 2.84) and V-J (64.00 ± 2.85) was significantly higher than other groups, in which Group V-J was the best (*P* < 0.05). There was no significant difference between the Group J (55.74 ± 2.53) and Group V (56.05 ± 3.39) (*P* > 0.05).

### Subjects’ attitude towards the inlay preparation operation of virtual simulation systems

**Fig. 9:**
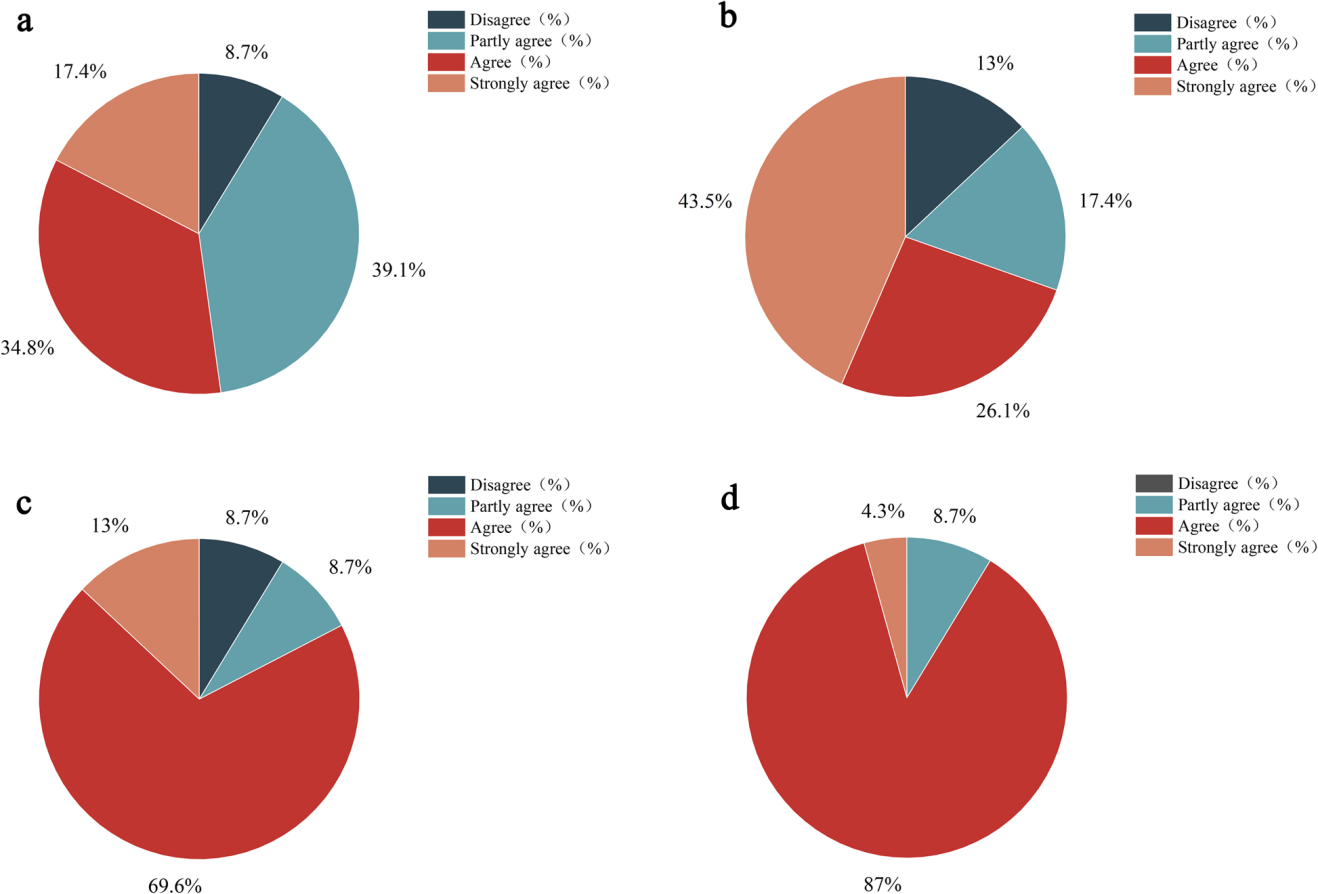
The attitude of inlay preparation operation of virtual simulation system. **a** Digital virtual simulation system is helpful to the improvement of dental preparation skills. Disagree: 8.7%, partly agree: 39.1%, agree: 34.8%, strongly agree: 17.4%. **b** Digital virtual simulation system is more difficult than traditional jaw simulation model training. Disagree: 13%, partly agree:17.4%, agree: 26.1%, strongly agree: 43.5%. **c** Digital virtual simulation system scores accurately. Disagree: 8.7%, partly agree: 8.7%, agree: 69.6%, strongly agree:13%. **d** Digital Virtual Simulation System has perfect assessment items. Disagree: 0%, partly agree: 8.7%, agree: 87%, strongly agree: 4.3%

A Likert scale was adopted by the subjects to subjectively evaluate the virtual simulation system. Table 3 shows the percentage of the number of people with different attitudes to the total number. Additionally, Fig. 9 (a), (b), (c), and (d) use a pie chart to show the proportion of different attitude choices in different questions. All study participants responded to the questionnaire inquiries. The questionnaire survey showed that most people think that the digital virtual simulation system has accurate scores and perfect assessment items, which helps improve dental preparation skills. Compared with the jaw model, its operation training was somewhat difficult. It may be because the virtual simulation system is a virtual image. Although it achieved a high degree of simulation for visual and tactile sense, it had a slightly worse physical sense compared to the jaw model, which made the training difficult for the subjects.

## Discussion

An inlay restoration is an important form of treatment for tooth defects. However, difficulty truly exists in clinical operation, particularly for complete beginners, because of its invasion and technical sensitivity [21]. The jaw model is a conventional means for dental experimental and preclinical skills training; whereas, this approach is higher in time-consumption and cost with few models. Therefore, it cannot simulate various cases in the clinical practice of real situations [3–6]. Fortunately, the virtual system can make up for the drawbacks of the jaw model and considerably meet the requirements of training. The main objective of this study was to explore the effect of the virtual-reality system in the preclinical teaching of inlay preparation to foster the preclinical ability and skills of undergraduates. This was done to investigate appropriate teaching approaches and propose new perspectives for reforming the traditional oral preclinical experimental teaching.

The experimental results showed that students using only the virtual system or jaw model gained significantly lower scores in both theoretical and operation exams than those who practiced under the plan combining the virtual system training and jaw model. This showed that pure virtual or traditional training methods did not achieve a satisfactory effect, indicating the strength of the combination mode. Using the virtual system separately can only simulate sensory and tactile stimuli during tooth preparation [22, 23]; however, it cannot simulate the actual clinical operation [24]. Conversely, using the jaw model training separately, students cannot experience the actual clinical tactile feeling and correct the errors in the depth and extent of tooth preparation in a timely manner [24]. In other words, the training on the jaw model allows the subjects to become more familiar with the clinical operation, and the training on the virtual system can help the subjects adapt to the sensory and haptic stimulation during the dental preparation [25]. Thus, their weaknesses were observed when separated, while their features and functions complement each other when combined. Further, the examination results of Groups J and V, where no significant difference between them was found, suggested that the traditional jaw model could not be eliminated or replaced in the preparation training for inlay restoration. VR was just a technology to deliver a learning technique for simulation that should not replace physical simulation like jaw models [26]; however, progress for improved assistance and combination in the future is required. Another explanation is that more people believe that the virtual simulation system can achieve the maximum effect after 4–5 times of training [27], which puts forward a new teaching idea for tooth preparation in inlay experiments. Compared to the other groups, Group V-J can better achieve the purpose of training, showing its significant training effect. The order of proper operation technique or standard first and then the practice on material objects may be used. The force feedback haptic system of the virtual system first enables the subjects to master specific skills [6, 28, 29]. Thereafter, the visual haptic feedback information is applied to the highly simulated jaw model for further operation training, which can enhance the students' understanding and command of the operation [6]. Additionally, sequential effects play an important role in learning and decision-making [29–32]. We found that Group V-J showed higher theoretical and operation scores than Group J-V, and the method of Group V-J was concluded as the best training mode in this study. In the process, students learned the basic theory first and acquired the operation stability based on proper technique and standard, and the operation training and assessment contained a specific amount of subjectivity. From this perspective, Group V-J, which first considered the construction and correction of the operation technique and standard of the students may improve comprehension among students, thereby advancing their subjective understanding of the operation method.

The results of the questionnaire survey can be concluded as follows: the training of the virtual system still cannot replace the jaw model, and there is good acceptance and enjoyment of virtual systems. Great affection was received towards the VR system, which opposes the findings of former studies [15]. While most participants have a positive attitude towards the virtual system according to the questionnaire survey, its critical weakness is that it cannot perform fulcrum exercises for the operator and cannot simulate the oral clinical operation process; thus, its degree of simulation is not as high as that of the jaw model [25, 28]. Current research on the application of virtual simulation systems in preclinical teaching has affirmed its effectiveness. In the dental preparation experiment of prosthetics, research shows that the virtual system used in the preclinical teaching of dental preparation is beneficial for improving the clinical skills of students [15, 17].

Previous studies did not emphasize the combination of virtual system training and jaw model training nor did they study the influence on the effect of the differences in the sequence of the two training modalities, and these reflect the strength of this research. The virtual system has a characteristic of immersion and interaction [3, 24, 33]; operators can get a sense of touch and operation feedback in the system [22, 23]. Although it builds a virtual operating environment and uses virtual operating tools to perform virtual operations on virtual patients, it can get real information feedback and technical improvement [34, 35]. As a teaching method, it provides repeatable, recorded, and computerised training, which typically requires no supervision [36], saving teacher resources and reducing the pressure of preclinical teaching. Despite the high initial cost [3], it requires fewer consumables while being used more frequently [6, 25]. Moreover, common and difficult cases in actual clinics can be simulated under virtual simulation conditions to improve the diagnosing and treatment skills of the operator by saving preclinical training time, improving performance, etc. [4, 36, 37]. Virtual simulation technology is not only suitable for preclinical teaching but also closely related to various branches of stomatology, and it has a wide range of applications in artificial-based medical fields, including dentistry [38–40].

Nevertheless, this study has some limitations. First, the study only enrolled a few undergraduates. Moreover, further studies are supposed to be utilized to explore the long-term effects of the virtual stimulation system, including the optimal application period for the teaching process. In this study, the assessment of tooth preparation was scored by clinical teachers, which had a specific degree of subjectivity, whereas the oral digital scanning technology was used to make the shape of the tooth preparation clear and provide a guarantee for the accuracy of the score [41, 42]. Consequently, the improvement of the virtual system with the advantage of jaw model, which is realizing the similarity in the training experience of jaw model with real clinical operations, may be one of the key research directions.

## Conclusion

These experimental results showed that virtual simulation training can facilitate the teaching effect of dental preparation in inlay experiments, and the teaching mode of the virtual simulation system followed by jaw model (Group V-J) was the best. Therefore, we advocate the combination of a virtual system and jaw model as a preclinical teaching, which is expected to open up new ideas of experimental teaching in stomatology and to be popularised.

## Supplementary Information

**Additional file 1.**

## Data Availability

The datasets used and/or analyzed during the current study are available from the corresponding author on reasonable request.

## Abbreviations

ANOVA: Analysis of variance
VR: Virtual reality

## References

[1] Johnson KS, Schmidt AM, Bader JD, Spallek H, Rindal DB, Enstad CJ (2020). Dental decision simulation (DDSim): Development of a virtual training environment. J Dent Educ..

[2] Kuric KM, Harris BT, Morton D, Azevedo B, Lin WS (2018). Integrating hinge axis approximation and the virtual facial simulation of prosthetic outcomes for treatment with CAD-CAM immediate dentures: A clinical report of a patient with microstomia. J Prosthet Dent..

[3] Buchanan JA (2001). Use of simulation technology in dental education. J Dent Educ..

[4] Samadbeik M, Yaaghobi D, Bastani P, Abhari S, Rezaee R, Garavand A (2018). The applications of virtual reality technology in medical groups teaching. J Adv Med Educ Prof..

[5] Liu L, Zhou R, Yuan S, Sun Z, Lu X, Li J (2020). Simulation training for ceramic crown preparation in the dental setting using a virtual educational system. Eur J Dent Educ.

[6] de Boer IR, Lagerweij MD, de Vries MW, Wesselink PR, Vervoorn JM (2017). The effect of force feedback in a virtual learning environment on the performance and satisfaction of dental students. Simul Healthc..

[7] Ganry L, Hersant B, Sidahmed-Mezi M, Dhonneur G, Meningaud JP (2018). Using virtual reality to control preoperative anxiety in ambulatory surgery patients: A pilot study in maxillofacial and plastic surgery. J Stomatol Oral Maxillofac Surg..

[8] de Juan-Ripoll C, Soler-Domínguez JL, Guixeres J, Contero M, Álvarez Gutiérrez N, Alcañiz M (2018). Virtual reality as a new approach for risk taking assessment. Front Psychol..

[9] Chen X, Hu J (2018). A review of haptic simulator for oral and maxillofacial surgery based on virtual reality. Expert Rev Med Devices..

[10] Nassar HM, Tekian A (2020). Computer simulation and virtual reality in undergraduate operative and restorative dental education: A critical review. J Dent Educ..

[11] Liu L, Zhou R, Yuan S, Sun Z, Lu X, Li J (2020). Simulation training for ceramic crown preparation in the dental setting using a virtual educational system. Eur J Dent Educ..

[12] Perry S, Bridges SM, Zhu F, Leung WK, Burrow MF, Poolton J (2017). Getting to the root of fine motor skill performance in dentistry: brain activity during dental tasks in a virtual reality haptic simulation. J Med Internet Res..

[13] Moglia A, Sinceri S, Ferrari V, Ferrari M, Mosca F, Morelli L (2018). Proficiency-based training of medical students using virtual simulators for laparoscopy and robot-assisted surgery: Results of a pilot study. Updates Surg..

[14] Perry S, Bridges SM, Burrow MF (2015). A review of the use of simulation in dental education. Simul Healthc..

[15] Reymus M, Liebermann A, Diegritz C (2020). Virtual reality: An effective tool for teaching root canal anatomy to undergraduate dental students-a preliminary study. Int Endod J..

[16] Vincent M, Joseph D, Amory C, Paoli N, Ambrosini P, Mortier É (2020). Contribution of haptic simulation to analogic training environment in restorative dentistry. J Dent Educ..

[17] Zafar S, Lai Y, Sexton C, Siddiqi A (2020). Virtual reality as a novel educational tool in pre-clinical paediatric dentistry training: Students' perceptions. Int J Paediatr Dent..

[18] Spitznagel FA, Scholz KJ, Strub JR, Vach K, Gierthmuehlen PC (2018). Polymer-infiltrated ceramic CAD/CAM inlays and partial coverage restorations: 3-year results of a prospective clinical study over 5 years. Clin Oral Investig..

[19] Chen Y, Chen D, Ding H, Chen Q, Meng X (2022). Fatigue behavior of endodontically treated maxillary premolars with MOD defects under different minimally invasive restorations. Clin Oral Investig..

[20] World Medical Association (2013). World Medical Association Declaration of Helsinki: ethical principles for medical research involving human subjects. JAMA..

[21] Becker M, Chaar MS, Garling A, Kern M (2019). Fifteen-year outcome of posterior all-ceramic inlay-retained fixed dental prostheses. J Dent..

[22] Yu H, Zhang CY, Zhang SH, Cheng H, Chen J (2017). Virtual simulation teaching centre in dental education: A report from Fujian Medical University. China. Chin J Dent Res..

[23] Dammerer D, Putzer D, Wurm A, Liebensteiner M, Nogler M, Krismer M (2018). Progress in knee arthroscopy skills of residents and medical students: A prospective assessment of simulator exercises and analysis of learning curves. J Surg Educ..

[24] Plessas A (2017). Computerized virtual reality simulation in preclinical dentistry: Can a computerized simulator replace the conventional phantom heads and human instruction?. Simul Healthc..

[25] Al-Saud LM, Mushtaq F, Allsop MJ, Culmer PC, Mirghani I, Yates E (2017). Feedback and motor skill acquisition using a haptic dental simulator. Eur J Dent Educ..

[26] Pottle J (2019). Virtual reality and the transformation of medical education. Future Healthc J..

[27] Dascal J, Reid M, IsHak WW, Spiegel B, Recacho J, Rosen B (2017). Virtual reality and medical inpatients: A systematic review of randomized, controlled trials. Innov Clin Neurosci..

[28] Wang D, Li T, Zhang Y, Hou J (2016). Survey on multisensory feedback virtual reality dental training systems. Eur J Dent Educ..

[29] Gulati S, Wasser T, Donato AA (2015). Order of curricular interventions in recognition of haematopathological images. Med Educ..

[30] Kooken J, Welsh ME, McCoach DB, Miller FG, Chafouleas SM, Riley-Tillman TC (2017). Test order in teacher-rated behavior assessments: Is counterbalancing necessary?. Psychol Assess..

[31] Ma T, Komarova NL (2019). Object-label-order effect when learning from an inconsistent source. Cogn Sci..

[32] Rey A, Le Goff K, Abadie M, Courrieu P (2020). The primacy order effect in complex decision making. Psychol Res..

[33] Kim M, Jeon C, Kim J (2017). A study on immersion and presence of a portable hand haptic system for immersive virtual reality. Sensors (basel)..

[34] Son SA, Kim JH, Seo DG, Park JK (2022). Influence of different inlay configurations and distance from the adjacent tooth on the accuracy of an intraoral scan. J Prosthet Dent..

[35] Kim JH, Son SA, Lee H, Kim RJ, Park JK (2021). In vitro analysis of intraoral digital impression of inlay preparation according to tooth location and cavity type. J Prosthodont Res..

[36] Plessas A (2017). Computerized virtual reality simulation in preclinical dentistry: Can a computerized simulator replace the conventional phantom heads and human instruction?. Simul Healthc..

[37] Makransky G, Bonde MT, Wulff JS, Wandall J, Hood M, Creed PA (2016). Simulation based virtual learning environment in medical genetics counseling: an example of bridging the gap between theory and practice in medical education. BMC Med Educ..

[38] Wang Q, Li C, Xie Z, Bu Z, Shi L, Wang C (2020). The development and application of virtual reality animation simulation technology: Take gastroscopy simulation system as an example. Pathol Oncol Res..

[39] Pereira D, Amelia-Ferreira M, Cruz-Correia R, Coimbra M (2020). Teaching cardiopulmonary auscultation to medical students using a virtual patient simulation technology. Annu Int Conf IEEE Eng Med Biol Soc..

[40] Pulijala Y, Ma M, Pears M, Peebles D, Ayoub A (2018). An innovative virtual reality training tool for orthognathic surgery. Int J Oral Maxillofac Surg..

[41] Moazami F, Bahrampour E, Azar MR, Jahedi F, Moattari M (2014). Comparing two methods of education (virtual versus traditional) on learning of Iranian dental students: A post-test only design study. BMC Med Educ..

[42] Ria S, Cox MJ, Quinn BF, San Diego JP, Bakir A, Woolford MJ (2018). A scoring system for assessing learning progression of dental students' clinical skills using haptic virtual workstations. J Dent Educ..

